# Cognitive Deficit and Aberrant Intrinsic Brain Functional Network in Early-Stage Drug-Naive Parkinson’s Disease

**DOI:** 10.3389/fnins.2022.725766

**Published:** 2022-02-25

**Authors:** Lan Zhang, Tao Yang, Yuping Chen, Denise Zheng, Dong Sun, Qiang Tu, Jinbai Huang, Junjian Zhang, Zezhi Li

**Affiliations:** ^1^Department of Neurology, The First Affiliated Hospital of Yangtze University, Jingzhou, China; ^2^Department of Neurology, Zhongnan Hospital of Wuhan University, Wuhan, China; ^3^Qingdao Mental Health Center, Qingdao University, Qingdao, China; ^4^McGovern Medical School, Houston, TX, United States; ^5^Department of Radiology, The First Affiliated Hospital of Yangtze University, Jingzhou, China; ^6^Department of Psychiatry, The Affiliated Brain Hospital of Guangzhou Medical University, Guangzhou, China; ^7^Guangdong Engineering Technology Research Center for Translational Medicine of Mental Disorders, Guangzhou, China

**Keywords:** Degree Centrality, cognitive deficit, Parkinson’s disease, resting-state functional magnetic resonance imaging (fMRI), early-stage drug-naive

## Abstract

**Background:**

Although cognitive deficit is a common non-motor symptom of Parkinson’s disease (PD), the mechanism and valid biomarkers of it have not been identified. To our best knowledge, this was the first study to investigate the intrinsic dysconnectivity pattern of whole-brain functional networks in early-stage drug-naive (ESDN) PD patients and its association with cognitive deficit of PD using voxel-wise Degree Centrality (DC) approach.

**Methods:**

A total of 53 ESDN PD patients and 53 healthy controls (HC) were recruited. Resting-state fMRI (rs-fMRI) data were acquired, and voxel-wise DC approach was applied. Electrophysiological testing at P300 amplitude was recorded. The Montreal Cognitive Assessment (MoCA) was conducted to evaluate cognitive performance.

**Results:**

ESDN PD patients had lower MoCA scores and P300 amplitudes, but higher P300 latency, than HC (all *p* < 0.0001). PD patients displayed higher DC in the right inferior frontal gyrus (IFG), left medial frontal gyrus (MFG) and left precentral gyrus (PreCG); but lower DC in the left inferior parietal lobule (IPL), left inferior temporal gyrus (ITG), right occipital lobe, and right postcentral gyrus (PoCG) (p_Bonferroni correction_ < 0.0001). Interestingly, the DC values of left MFG, right PoCG and right occipital lobe were negatively associated with P300 latency but positively associated with P300 amplitudes and MoCA scores (all p_Bonferroni correction_ < 0.0001).

**Conclusions:**

Our results indicate the cognitive deficit and abnormal intrinsic brain functional network in ESDN PD patients. The damage of Default Mode Network (DMN) may be contributes to the pathogenesis of cognitive dysfunction in ESDN PD.

## Introduction

Cognitive deficit is a common, non-motor symptom in the early to moderate stages of Parkinson’s disease (PD) (Aarsland et al., 2009), even in newly diagnosed PD patients without exposure to medication (Janvin et al., 2006; Aarsland et al., 2009). The incidence of cognitive deficit in early-stage, drug-naive (ESDN) PD patients is twice as high as that of the general population’s control subjects (Aarsland et al., 2009). With further progression of the disease, up to 80% of PD patients with mild cognitive deficit may develop PD dementia (PDD) (Hely et al., 2008). At present, the diagnosis of cognitive deficit mainly relies on the patient’s clinical symptoms and their cognitive scale evaluation results, lacking of objective examination results. Clinicians rarely pay attention to the cognitive function of ESDN PD patients for no obvious symptoms of cognitive impairment. Therefore, it is particularly important to identify cognitive deficit of PD patients early.

Currently, the pathophysiological mechanism of cognitive deficit in PD has not been elucidated, and no valid biomarkers have been identified, especially in early-stage PD patients. However, with the rapid development of functional magnetic resonance imaging (fMRI) technology in recent years, there is now a method for measuring brain changes in PD patients with cognitive deficit. Accumulating studies have shown abnormal default mode networks (DMN) (van Eimeren et al., 2009; Rektorova et al., 2012) and fronto-parietal networks (Tinaz et al., 2008; Lebedev et al., 2014) in PD patients with cognitive deficit. Previous evidence demonstrated that damage of the lobo-striatal circuit was the basis of cognitive and emotional disorders in PD patients (Owen, 2004). However, the results are still inconsistent, and most studies have not eliminated the confounding effect of drug treatment.

The development of graph theory-based methods provides a promising strategy to understand the characteristics of brain functional network. The Degree Centrality (DC) method, which is based on the degrees in the graph, could depict the importance of certain brain regions in the brain network by nodes. It also enables the investigation of these neural networks by detecting the number of instantaneous functional connectivity (FC) between one region and other brain region (Buckner et al., 2009). Notably, it was demonstrated that DC abnormalities can be observed in the early stages of several neuropsychiatric disorders, including schizophrenia (Wang et al., 2018), major depressive disorder (Zhang et al., 2011), and Alzheimer’s disease (Wang et al., 2013). Although the DC method can be effectively used to investigate brain functional activity changes of PD patients (Zhong et al., 2019), the DC abnormalities and its relationship with cognitive deficit in the ESDN PD patients has not been examined.

Because PD patients are prone to cognitive impairment and changes of resting-state brain function, we have a hypothesis that the ESDN PD patients also have cognitive impairment and brain function changes, and the brain function changes is related to cognitive function changes. To our best knowledge, this was the first study to investigate the intrinsic dysconnectivity pattern of whole-brain functional networks in ESDN PD and its association with cognitive deficit of PD through voxel-wise DC approach.

## Materials and Methods

### Study Participants

The study was approved by the Ethics Committee of the First Affiliated Hospital of Yangtze University. The procedure was carefully explained to all participants. Signed informed consent forms were obtained from all participants.

A total of 53 PD patients were recruited from the Department of Neurology, the First Affiliated Hospital of Yangtze University from May 2015 to May 2019. The inclusion criteria was as follows: (1) met the UK PD Society Brain Bank Criteria; (2) illness duration was less than 2 years, and Hoehn and Yahr (H&Y) stage ≤ 2 at baseline; (3) did not receive any anti-PD or antidepressant medications before fMRI scans; (4) without any apparent cognitive impairment [Mini-Mental State Examination (MMSE) scores > 24] and were right-handed; All subjects were free of dementia according to their MMSE scores; and (5) All subjects did not met the Diagnostic and Statistical Manual of Mental Disorders-Fifth Edition criteria for depression/anxiety disorder, who were diagnosed using the Structured Clinical Interview for the Diagnostic and Statistical Manual of Mental Disorders-Fifth Edition and HAMA scale and HAMD scores.

A total of 53 matched healthy controls (HC) without personal or family history of neuropsychiatric disorders were recruited from the community. Furthermore, no cerebral lesions and cerebral atrophy were found by MRI. The age, sex and education were comparable between patient and control groups (all *p* > 0.05).

### Cognitive Performance and Psychological Tests

All subjects were interviewed by neurologists. The Montreal Cognitive Assessment (MoCA) Scale was used to evaluate cognitive performance. Mini-Mental State Exam (MMSE) was applied to screen for dementia. Unified Parkinson’s Disease Rating Scale Part-III (UPDRS-III) was applied to evaluate motor symptoms. Hoehn and Yahr scale was applied to evaluate PD progression symptoms. The17-Hamilton Rating Scale for Depression (HDRS-17) and the Hamilton Anxiety Rating Scale (HAMA) were applied to assess depressive and anxiety symptoms, respectively. All raters were conducted by two neurologists with a correlation coefficient of more than 0.8.

### P300 Testing

P300 is an electrophysiological indicator for cognitive performance. The P300 amplitude reflects the capability of receiving external information and the amount of resources invested. The P300 latency represents cognitive performance and advanced thinking activity (Frodl et al., 2002). We recorded the P300 using the KeyPoint Brain Electrical Physiological Instrument. The subjects received short tone stimulation in both ears with a frequency of 1/s, a duration of 10 ms, and a sensitivity of 5 μV. Two sets of trigger and stimulus systems and two independent time windows were used to detect P300. The non-target stimulus (NT) intensity was 85 dB, and the frequency was 1,000 Hz. The target stimulus (T) intensity was 95 dB, and the frequency was 2,000 Hz. The target stimulus (T) appeared randomly and was interspersed throughout the non-target stimulus, accounting for 20% of total stimuli heard. The non-target stimulus accounted for the remaining 80% of total stimuli heard. The number of non-target stimulus was 8; the number of target stimulus was 4. All subjects had normal hearing.

### Imaging Acquisition

All image acquisitions were conducted by a practiced neuroradiologist via a Philips Achieva 3.0T MRI Scanner. The subjects remained motionless and stayed awake with their eyes closed, having as little thoughts as possible. All of these requisitions were to keep the subjects in a resting state (Zang et al., 2004). Firstly, normal T1-weighted and T2-weighted images were conducted to exclude obvious abnormal structures and brain atrophy after completing positional scanning. Resting-state functional images (rs-fMRI) were then obtained by scanning participants with echo-planar imaging sequence. The parameters were set as follows: 40 axial slices, matrix = 64 × 64, thickness/skip = 3/0.75 mm, echo time (TE) = 30 ms, repetition time (TR) = 2000 ms, flip angle = 90°, field of view (FOV) = 240 × 240 mm^2^, in-plane resolution = 3.75 × 3.75, and scan time = 8 min 6 s. Each condition comprised 240 functional volumes.

### Image Data Processing

The imaging data was preprocessed with the software packages of statistical parametric mapping (SPM8^1^) and Data Processing & Analysis for Brain Imaging (DPABI) V4.0^2^ running on Matlab 9.4 (The MathWorks, Inc., Natick, MA, United States). The covariates including age, sex, education level, HDRS-17 score, HAMA score and MMSE score were adjusted for in SPM8. We used the MRIcroN software convert image data in DICOM format into NIFTI format^3^. The first 10 volumes of fMRI images were discarded and the remaining 230 image data were performed slice timing, head motion corrections, and spatial normalization into the standard Montreal Neurological Institute (MNI) space and resampled to 3 × 3 × 3 mm^3^ voxels using DPABI. The participants were excluded for head motion (translation head motion larger than 1.5 mm, angular motion larger than 1.5) during the whole fMRI scan. Then the images were spatial smoothed using a Gaussian kernel of 8 mm full-width at half-maximum filter.

To reduce low frequency drift, the images were band-pass filtered (0.01 Hz < f < 0.08 Hz). To reduce the influence of increasing temperature from the MRI equipment, linear trend was removed. Finally, several nuisance variables were regressed out, including head motion parameters, signals of the brain white matter and cerebrospinal fluid. Final generated images went into DC analysis.

### Voxel-Based Degree Centrality (DC) Calculation

As has been reported in previous studies about DC^16^, we calculate the correlation coefficients between the time series of all pairs of gray matter voxels of the brain within the gray matter mask on the preprocessed rs-fMRI data using the Resting-State fMRI Data Analysis Toolkit (REST)^4^. The processing of the REST toolbox included the following procedures: (1) Import the preprocessed data through the button of input Data Directory; (2) Select Default Mask in the Mask option; (3) Fill Pearson’s correlation coefficient in the blank box of r(correlation) threshold as 0.25^9^; (4) Select the button of z-transform:subtract the mean and divide by the STD within the mask; and (5) Click the button of Run to perform DC calculation. We statistically analyzed the mean Z-score DC maps.

### Statistical Analysis

To examine the differences of DC value between the PD patients and health groups, we performed two sample *t*-test on the individual DC maps using DPABI V4.0. The resulting statistical map was set to uncorrected *p* < 0.001 and a minimum cluster size of 22 voxels (594 mm^3^), combined with a corrected threshold of *p* < 0.05. This was confirmed by using Monte Carlo simulations that were implemented in AlphaSim of AFNI^5^. We extracted the mean DC values of the clusters using the software REST V1.8.

Analyses of demographic and clinical data were done using the software SPSS 17.0 (SPSS Inc., Chicago, IL, United States). We used the method of two sample *t*-test to compare demographic and clinical data between the two groups. To detect the relationship between DC values and the clinical data of PD patients, we used Pearson correlation analysis.

## Results

### Demographics and Clinical Data

The demographics and clinical data are showed in Table 1. There were no significant differences in age, sex, education, HAMA score, HDRS score, and MMSE score between controls group and PD patients (all *p* > 0.05). The MoCA score and the P300 amplitudes of PD patients were significantly lower than those of controls group (p_Bonferroni correction_ < 0.0001), while the P300 latency of PD patients was significantly higher than that of controls group (p_Bonferroni correction_ < 0.0001).

**TABLE 1:** Demographic and clinical data of PD patients and healthy controls.

	PD patients (*n* = 53)	Healthy controls (*n* = 53)	*t*/x2	*p* value
Sex (male/female)	28/25	28/25	<0.0001	1
Age	54.35 ± 5.32	52.62 ± 6.45	1.506	0.14
Education level (years)	11.36 ± 4.56	12.14 ± 4.21	−0.91	0.36
HDRS-17 score	6.36 ± 2.88	5.85 ± 2.65	0.95	0.35
HAMA score	7.50 ± 3.85	6.73 ± 2.36	1.24	0.22
MMSE score	27.28 ± 1.37	27.77 ± 1.39	−1.83	0.07
MoCA score	26.44 ± 0.73	28.36 ± 0.55	−15.29	0.00
P300 amplitudes (μV)	10.12 ± 1.35	11.67 ± 1.87	−4.89	0.00
P300 latency (ms)	368.30 ± 15.93	355.97 ± 19.33	3.58	0.00
UPDRS-III score	14.78 ± 4.65	–	–	–
Hoehn and Yahr stage	1.42 ± 0.53	–	–	–
Age of onset	52.6 ± 3.67	–	–	–
Illness duration (years)	1.16 ± 0.68	–	–	–

*Data are expressed as mean ± SD. SD, standard deviation; PD, Parkinson’s disease; HDRS-17, 17 Hamilton Depression Rating Scale; HAMA, Hamilton Anxiety Rating Scale; MMSE, Mini-Mental State Exam; MoCA, Montreal Cognitive Assessment; UPDRS-III, Unified Parkinson’s Disease Rating Scale Part-III.*

### Voxel-Wise Degree Centrality Value of Parkinson’s Disease Patients and Healthy Controls

**FIGURE 1:**
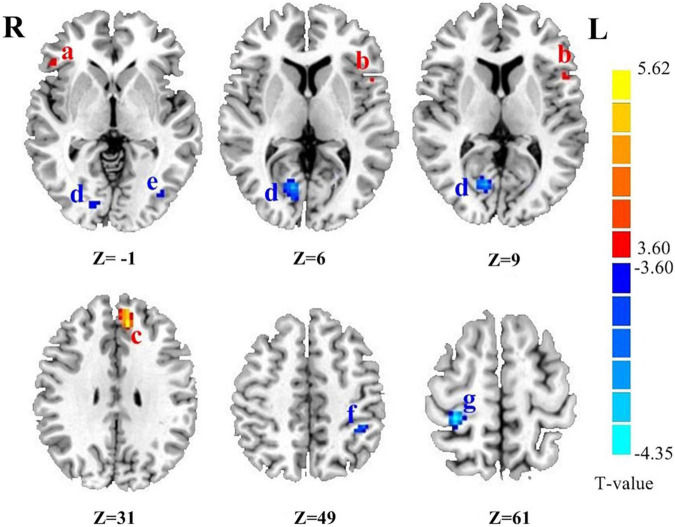
The brain regions with abnormal DC value in PD patients. **(a)** Right inferior frontal gyrus. **(b)** Left precentral gyrus. **(c)** Left medial frontal gyrus. **(d)** Right occipital lobe. **(e)** Left inferior temporal lobe gyrus. **(f)** Left inferior parietal lobule. **(g)** Right postcentral gyrus. Z is the coordinates of the Montreal Neurological Institute. The color scale indicates the magnitudes of *T*-values.

As shown in Table 2, compared with the controls group, PD patients had higher DC values in the left medial frontal gyrus (MFG), right inferior frontal gyrus (IFG) and left precentral gyrus (PreCG) (all p_Bonferroni correction_ < 0.0001). PD patients had lower DC values than HC in the left inferior parietal lobule (IPL), left inferior temporal gyrus (ITG), right occipital lobe and right postcentral gyrus (PoCG) (all p_Bonferroni correction_ < 0.0001). The brain regions with abnormal DC value in PD patients were shown in Figure 1.

**TABLE 2:** Brain regions with abnormal DC in PD patients.

Brain regions	Voxels ^a^	MNI coordinates (X,Y,Z)^b^	*t*-value ^c^	*p*-value
Left medial frontal gyrus	329	−10, 46, 31	5.316	<0.0001
Right inferior frontal gyrus	176	48, 30, −1	3.983	<0.0001
Left precentral gyrus	144	−52, 14, 9	3.942	<0.0001
Left inferior parietal lobule	128	−43, −42, 49	−3.764	<0.0001
Left inferior temporal lobe gyrus	133	−39, −74, −1	−3.715	<0.0001
Right occipital lobe	637	15, −74, 6	−4.053	<0.0001
Right postcentral gyrus	296	29, −35, 61	−4.278	<0.0001

*The threshold was set at p < 0.001 (AlphaSim). MNI, Montreal Neurological Institute. ^a^ Total number of voxels in each cluster. ^b^ Coordinates of the voxel of maximal statistical significance within each brain region. ^c^ t-value for the voxel of maximal statistical significance in each cluster.*

### Correlation Analysis Results

**FIGURE 2:**
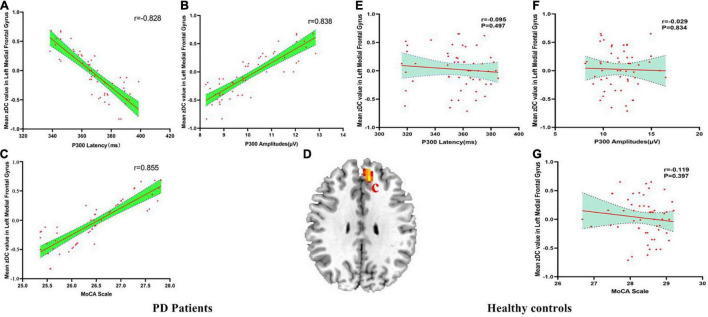
**(A–G)** The correlation results between mean zDC value in left medial frontal gyrus and P300 latency, P300 amplitudes and MoCA scale in PD patients and healthy controls. DC, Degree Centrality. The brain region in the red Alphabet c was left medial frontal gyrus.

There were negatively association between the DC values of left MFG and P300 latency (*r* = −0.828, p_Bonferroni correction_ < 0.0001), and there were positively association between the DC values of left MFG and P300 amplitudes (*r* = 0.838, p_Bonferroni correction_ < 0.0001) and MoCA scores (*r* = 0.855, p_Bonferroni correction_ < 0.0001) in ESDN PD patients (Figures 2A–C). No significant correlations between the DC value of left MFG and P300 latency (*r* = −0.095, p_Bonferroni correction_ = 0.497), P300 amplitudes (*r* = −0.029, p_Bonferroni correction_ = 0.834) and MoCA scores (*r* = −0.119, p_Bonferroni correction_ = 0.397) in HC were found (Figures 2E–G). Figure 2D showed the abnormal DC value in left MFG of the brain.

**FIGURE 3:**
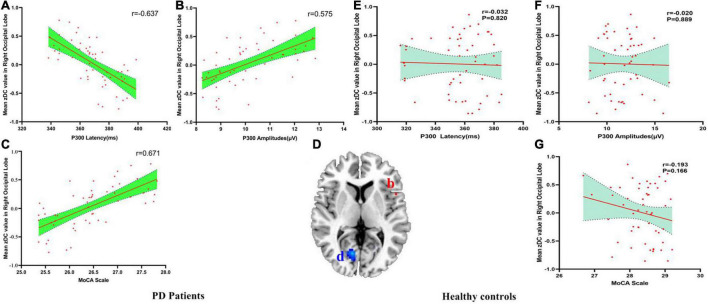
**(A–G)** The correlation results between mean zDC value in right occipital lobe and P300 latency, P300 amplitudes and MoCA scale in PD patients and healthy controls. DC, Degree Centrality. The brain region in the red Alphabet b was left precentral gyrus. The brain region in the blue Alphabet d was right occipital lobe.

There were negatively association between the DC values of right occipital lobe and P300 latency (*r* = −0.637, p_Bonferroni correction_ < 0.0001), and there were positively association between the DC values of right occipital lobe and P300 amplitudes (*r* = 0.575, p_Bonferroni correction_ < 0.0001) and MoCA scores (*r* = 0.671, p_Bonferroni correction_ < 0.0001) in PD patients (Figures 3A–C). No significant correlations between the DC value of right occipital lobe and P300 latency (*r* = −0.032, p_Bonferroni correction_ = 0.820), P300 amplitudes (*r* = −0.020, p_Bonferroni correction_ = 0.889) and MoCA scores (*r* = −0.193, p_Bonferroni correction_ = 0.166) in HC were found (Figures 3E–G). Figure 3D showed the abnormal DC value in right occipital lobe of the brain.

**FIGURE 4:**
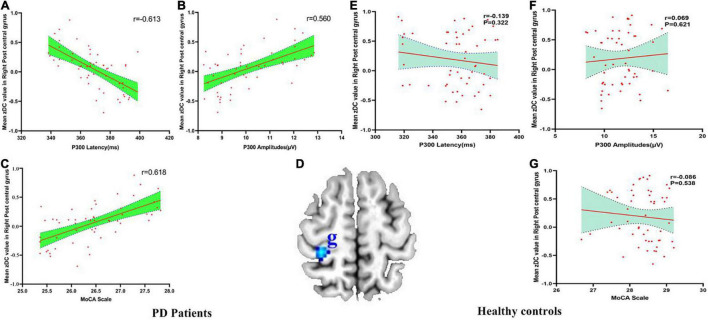
**(A–G)** The correlation between mean zDC value in the right postcentral gyrus and P300 latency, P300 amplitudes and MoCA scale in PD patients and healthy controls. DC, Degree Centrality. The brain region in the blue Alphabet g was right postcentral gyrus.

There were negatively association between the DC values of right PoCG and P300 latency (*r* = −0.613, p_Bonferroni correction_ < 0.0001), and there were positively association between the DC values of right PoCG and P300 amplitudes (*r* = 0.560, p_Bonferroni correction_ < 0.0001) and MoCA scores (*r* = 0.618, p_Bonferroni correction_ < 0.0001) in PD patients (Figures 4A–C). No significant correlations between the DC value of right PoCG and P300 latency (*r* = −0.139, p_Bonferroni correction_ = 0.322), P300 amplitudes (*r* = 0.069, p_Bonferroni correction_ = 0.621) and MoCA scores (*r* = −0.086, p_Bonferroni correction_ = 0.538) in HC were found (Figures 4E–G). Figure 4D showed the abnormal DC value in right PoCG of the brain.

There were no associations between the DC values of the right IFG, left PreCG, left IPL, left ITG and all the clinical characteristics including age, disease course, education, onset age, HDRS score, HAMA score, MMSE score, UPDRS-III score, and H&Y score (all *p* > 0.05).

## Discussion

The main results of this study were as follows: (1) MoCA score and P300 amplitude decreased and latency prolonged in PD patients compared with HC; (2) PD patients had higher DC values in the left MFG, right IFG, and left PreCG, but lower DC values in the left IPL, left ITG, right occipital lobe, and right PoCG compared to HC; and (3) the DC values of left MFG, right PoCG, and right occipital lobe were negatively associated with P300 latency, but positively associated with P300 amplitudes and MoCA scores in PD patients.

Our results showed that ESDN PD patients had lower cognitive performance than HC, which suggests that early stage PD patients who have never been exposed to medicine already have cognitive deficit. Furthermore, ESDN PD patients displayed decreased P300 amplitude and prolonged P300 latency, which indicates a reduced ability to understand, analyze, and respond to extrinsic information. These electrophysiological data revealed the cognitive deficit in ESDN PD patients.

The DMN is the core of the whole brain network in resting state, involving future imagination, episodic memory, emotional regulation, executive function, and other cognitive abilities (Raichle et al., 2001; He et al., 2014). Studies have found that the DMN was involved in the neural mechanism underlying cognitive deficit in PD patients (Hou et al., 2017). The greater the DC value, the more complete and faster the brain network connection and the more effective information resource utilization and information transmission (Zuo et al., 2012). In this study, the frontal lobe, which is the core region of the DMN, had brain regions with increased DC. It is well established that the frontal lobe is an important locus of mental activity as it is closely related to cognitive function, emotion, and thinking. Among the brain regions with decreased DC, the IPL, ITG and occipital lobe also belong to the DMN. Previous studies have shown lower FCs of the DMN in PD patients, which was associated with cognitive dysfunction of PD patients (Baggio et al., 2014, 2015; Hou et al., 2017). Regarding the pathogenesis of cognitive dysfunction in PD patients, some researchers believe that patients with abnormal function of the frontal-striatum displayed dysfunction in attention, memory, executive function, and speech fluency. Patients with posterior cortical deficit displayed visual, spatial, and memory abnormalities, such as naming and repetition (Jellinger, 2010; Kehagia et al., 2010). Thus, the Dual Syndrome Hypothesis about cognitive deficit in PD came into nascence: the dysfunction of the frontal-striatum dopaminergic system is associated with executive function deficit, whereas the dysfunction of the occipital, parietal, and temporal lobes’ cholinergic system is associated with the deficit of visuospatial function, memory, and attention (Kehagia et al., 2013). In this study, we found increased DC in the frontal lobe and decreased DC in the posterior cortex, both of which are consistent with previous studies and hypothesis (Jellinger, 2010; Kehagia et al., 2010; Baggio et al., 2014, 2015; Hou et al., 2017).

The MFG and IFG are key nodes in emotional regulation and cognitive control circuits (Roberts et al., 2017). The dysfunction of these brain regions already existed before significant changes in cognitive function, which resulted in the functional change of the DMN (Hou et al., 2017). Previous evidence also showed that the FC between the MFG, IFG, and other brain regions of the whole brain were weakened in PD patients (Olde Dubbelink et al., 2014). The PreCG is the main motor cortex in the brain and is responsible for the initiation and execution of movement. When performing the same motor task, PD patients needed to invest more neural activity in the PreCG to complete the nerve impulse of motor execution, which indicated that PD patients had dysfunction in the PreCG (Haslinger et al., 2001). Furthermore, the dysfunction in the PreCG and prefrontal cortex cause an absence of active movement and thinking in PD patients (Caproni et al., 2013). The non-motor symptoms of PD patients such as depression, cognitive dysfunction, and executive dysfunction may be associated with dysfunction of the PreCG and prefrontal cortex (Caproni et al., 2013). Therefore, the PreCG is thought to playa critical role in the executive ability of PD patients. Dysfunction of the PreCG will lead to difficulty with motor thoughts, motor execution, and task initiation (Caproni et al., 2013; Hou et al., 2017).

Some reports also demonstrated that PD patients with cognitive deficit had decreased gray matter volume (GMV) in the bilateral frontotemporal region, MFG, IFG, and cingulate gyrus (Sang et al., 2014). The loss of GMV in these brain regions was associated with cognitive deficit (Sang et al., 2014). This could be explained by a decrease of GMV leading to a decrease of FC and efficiency of brain network connection in PD with cognitive deficit (Baggio et al., 2014, 2015). However, contrary to previous reports, our results showed an increased DC in the frontal lobe region. Since all patients in this study were ESDN PD patients, they may have a compensatory response to the dysfunction of DMN and frontal-striatum in the brain, which sustains their executive function, emotional control, working memory, and other cognitive functions in a transient state of balance. So we have a hypothesis that the compensatory mechanisms exist in certain brain regions in ESDN PD patients. The increased DC value in the frontal lobe further indicates that the frontal lobe plays a key role in the network of cognitive function in PD patients with cognitive deficit. Therefore, increased DC in the frontal lobe may be a characteristic imaging marker of PD patients with cognitive deficit. However, further longitudinal research is needed to clarify this hypothesis.

The IPL is an integral part of the DMN, relating to pain perception, touch, taste, cognition, and spatial sensory processing. Neuroimaging studies have shown that the IPL was involved in a wide range of behavioral and sensory cognitive activities (Igelstrom and Graziano, 2017). The structural MRI study revealed that early onset PD patients had a wide range of GMV loss in the frontal, parietal, and temporal lobes, as well as decreased intensity of neural activity in these brain regions (Sang et al., 2014). Yang found that there was increased regional homogeneity (ReHo) in parietal lobules, but decreased ReHo in the PoCG in the early stages of PD, which suggests that increased ReHo was the compensatory response to the decreased functional activity of the PoCG (Yang et al., 2013). Other studies reported that the mean amplitude of low-frequency fluctuations (mALFF) of PD patients was significantly increased in the bilateral prefrontal lobe, parietal lobe, and lateral cortex of the temporal lobe (Liu et al., 2009). Our study showed a decreased DC in the left IPL, which is consistent with the developmental trend of cognitive deficit in PD patients. However, the subtype of cognitive deficit may be different from that of the selected patients in previous studies, resulting in different brain regions for compensation (Mallol et al., 2007).

The PoCG is an important sensory brain region that is mainly responsible for integrating various somatosensory stimuli information to achieve correct recognition of objects and external stimuli. It also accepts expressive projections that are closely related to emotional expression and social cooperation. Our study found decreased DC in the PoCG, suggesting a decrease in the ability to transmit information in the whole brain network, which was consistent with previous reports. For example, previous studies have found that the FC between the right PoCG, right PreCG, right precuneus, and the putamen were decreased in early onset PD patients without medication. These decreased FC were then associated with cognitive dysfunction (Liu et al., 2009; Sang et al., 2014). A meta-analysis emphasized that the left PoCG plays a critical role in non-motor symptoms of PD, and it may become a potential target for clinical intervention (Ji et al., 2018).

In this study, we found decreased DC in the right occipital lobe and left ITG, suggesting that PD patients already had visual-related cognitive dysfunction and episodic memory disorders. Previous evidence has shown that the right occipital lobe is related to spatial recognition of visual symbols, integration of visual information, and perception (Ramirez-Ruiz et al., 2007). The occipital lobe and lingual gyrus are involved in visual recognition, language, movement, abstract concepts, and consolidation of episodic memory (Kukolja et al., 2016). The increased ReHo value in the occipital lobe and temporal lobe in PD patients with sleep behavior disorder indicated the enhanced coordination and consistency of the neural activities in these two brain regions (Wang et al., 2010). A PET study found decreased metabolism in the occipital and temporal lobes in early-onset PD patients (Wu et al., 2014). These results suggest that there may be a synergistic effect between the occipital and temporal lobes in visual information processing in PD patients, which may be related to the termination of the visual radiation located in the temporal lobe. Some studies have also found that the reduction of FC in the occipital and parietal lobes was a key pathophysiological factor for the cognitive decline of PD patients with cognitive deficit (Jellinger, 2010; Baggio et al., 2014; Olde Dubbelink et al., 2014). Structural MRI studies have reported a decreased GMV, mainly in the occipital, temporal, and frontal lobes, in PD patients with cognitive deficit (Ramirez-Ruiz et al., 2007). Furthermore, PD patients with cognitive deficit have cortical atrophy in the left temporal cortex (Janvin et al., 2006). The decrease of GMV caused the decline of functional activity and efficiency of the FC in the corresponding brain regions, which lead to the decreased DC value.

In this study, we investigated the correlation between the DC values in all abnormal brain regions and clinical characteristics. We found that the DC values of the left MFG, the right PoCG, and the right occipital lobe were positively associated with MoCA scores and P300 amplitudes, but negative correlated with P300 latency. Our results indicated that the efficiency of brain FC in the frontal lobe, PoCG, and occipital lobe were associated with the cognitive function of PD patients, which was consistent with previous studies (Ramirez-Ruiz et al., 2007; Caproni et al., 2013; Yang et al., 2013; Baggio et al., 2014; Hou et al., 2017; Ji et al., 2018). We did not find a correlation between the DC values and age, education, age of onset, course of disease, HDRS score, HAMA score, MMSE score, UPDRS-III score, and H&Y score. The results in this study of ESDN PD patients indicated that the pathological characteristics of the natural disease process of PD patients impacts cognition, rather than the secondary changes caused by long-term anti-PD treatment or the neurotoxic damage to the brain caused by the chronic disease course. This correlation provides a reference and a new perspective for revealing the pathogenesis of cognitive deficit in PD patients.

There were several limitations of this study. This was a cross-sectional study, but PD is a progressive disease; thus, the dynamic changes of the DC value of these abnormal brain regions cannot be defined at present. Further longitudinal studies using multimodal neuroimaging techniques are needed to better understand the pathologic mechanisms in-depth. Further studies can also expand the sample size to explore the mechanism of brain dysfunction in patients with different subtypes and stages.

## Conclusion

This study indicates that cognitive deficit and abnormal intrinsic brain functional network exists in ESDN PD patients. The damage of the DMN may be contributes to the pathogenesis of cognitive dysfunction in ESDN PD.

## Data Availability Statement

The raw data supporting the conclusions of this article will be made available by the authors, without undue reservation.

## Ethics Statement

The studies involving human participants were reviewed and approved by the Ethics Committee of the First Affiliated Hospital of Yangtze University. The patients/participants provided their written informed consent to participate in this study.

## Author Contributions

JZ, LZ, and ZL: conception and design. JZ, DS, QT, and JH: administrative support. LZ, TY, and QT: provision of study materials or patients. LZ, TY, DS, and JH: collection and assembly of data. LZ and TY: data analysis and interpretation. ZL and YC: literatures collection and data clean. JZ, LZ, TY, ZL, and DZ: manuscript writing. All authors final approval of manuscript.

## Conflict of Interest

The authors declare that the research was conducted in the absence of any commercial or financial relationships that could be construed as a potential conflict of interest.

## Publisher’s Note

All claims expressed in this article are solely those of the authors and do not necessarily represent those of their affiliated organizations, or those of the publisher, the editors and the reviewers. Any product that may be evaluated in this article, or claim that may be made by its manufacturer, is not guaranteed or endorsed by the publisher.

## Footnotes

1
http://www.fil.ion.ucl.ac.uk/spm/software/spm8

2
http://rfmri.org/dpabi

3
http://www.mccauslandcenter.sc.edu/mricro/mricron/

4
http://www.restfmri.net

5
http://afni.nimh.nih.gov

## Abbreviations

DC: Degree Centrality
DMN: default mode networks
ESDN: early-stage drug-naive
FC: functional connectivity
HAMA: Hamilton Anxiety Rating Scale
HDRS-17: The17-Hamilton Rating Scale for Depression
IFG: inferior frontal gyrus
IPL: inferior parietal lobule
ITG: inferior temporal gyrus
mALFF: mean amplitude of low-frequency fluctuations
MFG: medial frontal gyrus
MMSE: Mini-Mental State Exam
MoCA: Montreal Cognitive Assessment
PD: Parkinson’s disease
PoCG: postcentral gyrus
PPD: Parkinson’s disease dementia
PreCG: precentral gyrus
ReHo: regional homogeneity
rs-fMRI: resting-state magnetic resonance imaging
UPDRS-III: Unified Parkinson’s Disease Rating Scale Part-III.
